# Novel Frontiers of Treatment for Advanced Gastric or Gastroesophageal Junction Cancer (GC/GEJC): Will Immunotherapy Be a Future Direction?

**DOI:** 10.3389/fonc.2020.00912

**Published:** 2020-07-21

**Authors:** Rilan Bai, Naifei Chen, Tingting Liang, Lingyu Li, Zheng Lv, Xiaomin Lv, Jiuwei Cui

**Affiliations:** ^1^Cancer Center, The First Hospital of Jilin University, Changchun, China; ^2^Department of Neurology, The First Hospital of Jilin University, Changchun, China

**Keywords:** gastric or gastroesophageal junction cancer, immunotherapy, combination therapy, immune related adverse events, biomarkers

## Abstract

Considering the limited progress of chemotherapy and targeted therapy in improving the generally disappointing outcomes of advanced gastric or gastroesophageal junction cancer (GC/GEJC), immunotherapies have been gradually developed and advanced into novel frontiers of treatment for advanced GC/GEJC. Nevertheless, the response to immunotherapy was not always satisfactory, and the emergence of resistance was unavoidable. These factors prompt the development of different combination therapies and predictive and prognostic biomarkers of efficacy to improve the outcomes of patients with advanced GC/GEJC and to overcome drug resistance. This article discusses the advances of immune monotherapy, multiple current and ongoing clinical trials of immune combination therapy, immune-related adverse events, and various biomarkers in GC/GEJC.

Gastric or gastroesophageal junction cancer (GC/GEJC) is the third most common cause of cancer deaths worldwide, and the incidence ranks fifth, 63% of which show locally advanced or metastatic disease (1). Considering the limited progress of traditional therapy, like chemotherapy and anti-Human epidermal growth factor receptor-2 (HER-2) therapy in improving the generally disappointing outcomes (2), and the genetic complexity and heterogeneity of GC/GEJC, immunotherapies have gradually been developed and advanced into novel frontiers of treatment for advanced GC/GEJC, entirely revolutionizing the therapeutic landscape in the last 10 years. Nowadays, a number of clinical trials with immunotherapies have been conducted or are ongoing. These clinical trials involve cancer vaccines [such as, dendritic cell (DC) vaccine, melanoma-associated antigen 3 (MAGE-3) peptide vaccine], adoptive cell therapies [such as cytokine-induced killer (CIK) cells, DC-CIK, chimeric antigen receptor (CAR)-T cell therapy], and immune checkpoint inhibitor (ICI) therapies. Some of these therapies have been approved for the treatment of advanced GC/GEJC, indicating the expanding range and potential of immunotherapy applications. Although the response obtained from immunotherapy in patients with GC/GEJC adenocarcinoma is only 10–20%, and the potential of drug resistance and rapid disease progression is likely, the exploration of mechanisms of resistance to immunotherapy, of effective immune combination therapy strategies, and of predictive and prognostic biomarkers is essential for issues in oncology. This article discusses advances of immune monotherapy, multiple current and ongoing clinical trials of immune combination therapy, immune-related adverse events (irAEs), and various biomarkers in GC/GEJC.

## Rationale for the Use of Immunotherapy in the Treatment of GC/GEJC

Landmark analyses by the Cancer Genome Atlas (TCGA) in 2014 proposed classifications based on comprehensive genomic profiling for four subtypes of gastric cancer (GC) (3): Epstein–Barr virus (EBV, 8%) infection, microsatellite instability (MSI) (22%), genomic stability (20%), and chromosomal instability (CIN) (50%). The EBV subtype GC is characterized by a high incidence of DNA hypermethylation and amplification of CD274 [encoding programmed death-ligand 1 (PD-L1)] and PGD1LG2 (encoding PD-L2). An increased expression of PD-L1/2 that were evaluated in mRNA from EBV-positive GCs in the TCGA cohort characterizes their immune profile, which is known to have prominent stromal lymphoid infiltrates and a high density of tumor infiltrating lymphocytes (TILs), establishing a balance between host immune evasion mediated by PD-L1/2 overexpression and host immune responses (4). Therefore, the EBV subtype is a promising choice for ICI therapy in GC. The ongoing phase II/III clinical trials **(NCT02488759** and **Checkmate-358)** are also evaluating the efficacy of nivolumab in EBV-positive GC. Chronic EBV infection can trigger Th1 antiviral responses which lead to antitumor responses, such as the induction of IFN-γ production (3). The MSI subtype GC has high mutation load, TILs, and neoantigen presentation of DCs and macrophages (3). Therefore, EBV-positive and MSI phenotype GCs display unique immune characteristics that may be suitable targets for immunotherapy (5–7). A comprehensive analysis of the molecular characteristics of 295 gastric adenocarcinomas shows that about 34% of GCs show a relatively high mutation load, including MSI-H (8). In addition, the level of TILs and a high expression of CD3, CD8, and C45RO in patients with GC have a certain predictive value of patient prognosis. Patients with TILs highly expressing a combination of these three markers showed a longer overall survival (OS) than those with low expression (9), suggesting that GC might be a better target disease for ICIs.

## Clinical Advances of Immune Monotherapy in GC/GEJC

### Cancer Vaccines

Cancer vaccines take advantage of antigens associated with tumor cells such as proteins overexpressed in tumor cells, cancer-testis antigens (CTAs), protein products of oncogenes, and heat-shock protein complexes (10), which may be recognized as foreign by the host adaptive immune system and trigger antitumor immune responses (11). MAGE-3 peptide vaccine acted as an adjuvant and was used to enhance an antitumor immune response resulting in a successful regression of tumor growth in a mouse model of GC (12). HER-2^+^ cancer is an example where overexpressed proteins have been exploited for vaccination (12, 13). DCs, stimulated with HER-2 peptides, which were capable of inducing antitumor immunity against HER-2^+^ GC, were developed as vaccines, and were evaluated in a phase I trial (13). NY-ESO-1 is a CTA expressed in gastroesophageal neoplasms. A phase I trial assessed the efficacy of NY-ESO-1 vaccine in tumors where 9 out of 10 patients with gastroesophageal cancer had an enhanced antibody response, and all patients had an increase in antigen-responsive CD4 and CD8 T cells (14). A peptide vaccine consisting of three different human leukocyte antigen (HLA)-A24-conjugated CTAs was assessed in a phase II clinical trial following promising phase I trial results (15). In cancer cells, heat shock proteins (HSP), acting as tumor rejection antigens, can form protein complexes with various deranged intracellular proteins and induce CD4^+^ and CD8^+^ T cell responses, suggesting that vaccines against HSP will play a role in immunotherapy for GC (16).

### Adoptive Cell Therapies

Adoptive cell therapies (ACTs) may use autologous lymphocytes that have been isolated from the tumor itself or from the blood and manipulated *in vitro* to enhance their activity by expressing particular T-cell receptors or CARs against target antigens (17). CAR-T GC patients received immunotherapy with EAALs that were stimulated by the IL-2 or anti-CD3 inhibitor. As a result, significantly longer OS was observed in the treatment group (18, 19). In GC, CAR-T therapy against four major antigens is currently being tested in clinical trials. First, HER-2 gene amplification has been reported in 1/3 of GCs. A trial of anti-HER-2 CAR-T therapy aiming to study the adverse effects in patients with advanced HER-2^+^ GC/GEC is ongoing **(NCT02713984)**. Next, carcinoembryonic antigen (CEA) is overexpressed in gastrointestinal tumors where its overexpression indicates poor prognosis in GC (20). A trial investigating the efficacy of anti-CEA CAR-T cell therapy in advanced CEA^+^GC has been initiated **(NCT02349724)**. Third, anti-MUC1 CAR-T cells are also being studied in patients with advanced MUC1^+^ GC/GEC **(NCT02617134)**. Finally, CAR-T therapy against epithelial cell adhesion molecule (EpCAM) is under trial **(NCT03013712)**. These trials are currently recruiting patients, and data on the antitumor efficacy and survival time of CAR-T cells in patients with advanced GC/GEC will be collected. However, available clinical trial data suggest that GC patients respond poorly to ACTs and there are insufficient ongoing trials assessing ACTs, reflecting the disappointing results. The reason for their poor response rate may be the induction of immune tolerance in adoptive cells. Therefore, combination therapies targeting multiple mechanisms of tumor-mediated immunomodulatory may need to be developed to overcome the poor efficacy seen in ACTs alone.

### ICI Monotherapy in GC/GEJC

Recently, immunotherapy with antibodies that inhibit PD-1/PD-L1 interaction has emerged as a new treatment option in the field of GC. Following the results from the Phase Ib Keynote012 study (21) and from the phase II Keynote-059 cohort 1 (22), the U.S. Food and Drug Administration (FDA) has approved pembrolizumab for third-line treatment of PD-L1^+^ [combined positive score (CPS) ≥ 1%] recurrent or metastatic GC/GEJC adenocarcinoma (22–25). However, the phase III Keynote-061 study (26) did not show significant survival benefits when pembrolizumab was used as a second-line treatment for PD-L1^+^ advanced GC, but improvement of OS, better efficacy, and fewer treatment related adverse events (TRAEs) were found in patients with ECOG 0, PD-L1 CPS ≥ 10, or MSI-H. Subsequently, phase III Keynote-062 (27) showed survival benefits in patients with PD-L1^+^, especially in PD-L1 CPS ≥ 10, making pembrolizumab possible as a first-line treatment. As for nivolumab, based on the results of the Phase III ATTRACTION-02 study (28), many regions approved nivolumab for the treatment of unresectable advanced or recurrent GC that progresses after chemotherapy, regardless of PD-L1 expression. Subsequent results in the Phase I/II Checkmate-032 study also confirmed survival benefit with nivolumab in the third-line setting (29). Due to the encouraging results from the JAVELIN Phase I trial (30) with avelumab, two randomized controlled phase 3 trials for avelumab are currently underway: JAVELIN 300 **(NCT02625623)** (31, 32) and JAVELIN 100 **(NCT02625610)** (33, 34). Disappointingly, the results of the JAVELIN 300 trial recently failed to reach its primary endpoint OS in order to consider avelumab as a third-line treatment option for advanced GC/GEJC adenocarcinoma that did not test for PD-L1. On the other hand, JAVELIN 100 is ongoing. Overall, there are still many trials being conducted to explore the effectiveness of immune monotherapy in GC. The Keynote 063 trial **(NCT03019588)** is comparing the efficacy of treatment with pembrolizumab vs. paclitaxel in Asian PD-L1^+^ patients with advanced GC who did not respond to any combination treatment containing a fluoropyrimidine and platinum agent. The ongoing phase II/III clinical trials **(NCT02488759** and **Checkmate-358)** are also evaluating the efficacy of nivolumab in EBV-positive GC. As for other PD-L1 inhibitors, for example, a phase Ib/II study in patients with advanced GC/GEJC is currently underway to test the role of durvalumab and tremelimumab as a second- or third-line single-agent and combination therapy **(NCT02340975)** (35).

At present, the anti-cytotoxic T-lymphocyte-associated protein 4 (CTLA-4) antibody, ipilimumab, did not reach the expected endpoint of improved progression free survival (PFS) and OS in advanced GC/GEJC adenocarcinoma (NCT01585987) (36). A phase II trial investigated tremelimumab as a second-line treatment in patients with metastatic gastric and esophageal adenocarcinoma. The objective response rate (ORR) was only 5%, but there was a clinical benefit with evidence of stable disease (SD) in 4 of the 18 patients enrolled, and one patient showed a durable response, obtaining 32.7 months of treatment (37). Currently, the efficacy of CTLA-4 inhibitor monotherapy is not clear, thus they are only used in clinical trials in combination with other agents, such as programmed death-1 (PD-1)/PD-L1 inhibitors.

The summary of ICI monotherapies in GC/GEJC is described in Table 1. Despite many encouraging results, most patients remain unresponsive to immunotherapy, manifesting primary resistance, or the emergence of an acquired resistance phenomena in initial responders after a period of treatment. Our understanding of the mechanisms of tumor resistance to immunotherapy involving tumor-intrinsic factors (such as lack of tumor antigen expression, loss of HLA expression, and alterations of signaling pathways) and tumor-extrinsic factors (such as local tumor microenvironment like immunosuppressive cells and molecules, and host-related factors like age, gender, intestinal flora) continue to expand and deepen (38), but the issue of tumor resistance remains complex and difficult to overcome. Therefore, multiple studies of immunotherapy in combination with other treatments are underway.

**Table 1:** The summary of ICI monotherapies in GC/GEJC.

**Agent**	**Clinical trial**	**Line**	**Phase**	**Outcomes**	**Significance**
Pembrolizumab	Keynote012	Terminal-line	Phase Ib	Safe and effective in PD-L1+ advanced GC	FDA approves pembrolizumab for third-line treatment of PD-L1+ (CPS ≥ 1%) recurrent or metastatic GC/GEJC adenocarcinoma.
	Keynote-059	Third-line	Phase II	PD-L1+ patients had higher response rates than negative patients	
	Keynote-061	Second-line	Phase III	Did not show significant survival benefits in mOS and mPFS of PD-L1+ advanced GC	Improvement of OS, better efficacy, and fewer TRAEs were found in patients with PD-L1 CPS ≥ 10 and MSI-H.
	Keynote-062	First-line	Phase III	Had survival benefits in patients with PD-L1+, especially in PD-L1 CPS ≥ 10	It makes pembrolizumab possible as a first-line treatment
	Keynote 063	Second-line	Phase III	Ongoing	–
Nivolumab	ATTRACTION-02	Third-line	Phase III	All patients could benefit from OS regardless of PD-L1 expression	Many regions approve nivolumab for the treatment of unresectable advanced or recurrent GC regardless of PD-L1 expression
	Checkmate-032	Third-line	Phase I/II	Had potential advantages over chemotherapy	–
	NCT02488759, Checkmate-358	–	Phase II/III	Ongoing	–
Avelumab	JAVELIN	First-line or second-line	Phase I	ORR, DCR, mPFS, and mOS had improved.	Encouraging results facilitate phase III studies
	JAVELIN 300	Third-line	Phase III	Failed to reach its primary endpoint OS recently	–
	JAVELIN 100	First-line maintenance	Phase III	Ongoing	–
Durvalumab and tremelimumab	NCT 02340975	Second- or third-line	Phase Ib/II	Ongoing	–
Ipilimumab	NCT01585987	First-line	Phase II	Did not reach expected endpoint of improved PFS and OS	Currently, the efficacy of CTLA-4 inhibitor monotherapy is not clear

*ICI, immune checkpoint inhibitor; DCR, disease control rate; FDA, Food and Drug Administration; GC, gastric cancer; GC/GEJC, gastric or gastroesophageal junction cancer; OS, overall survival; ORR, objective response rate; PFS, progression free survival; PD-L1, programmed death-ligand 1; CPS, combined positive score; TRAEs, treatment related adverse events; MSI-H, microsatellite instability-high; CTLA-4, cytotoxic T-lymphocyte-associated protein 4*.

## Clinical Advances of Immunotherapy in Combination With Other Therapies in GC/GEJC

Considering the poor efficacy of immunotherapy as a single agent, as well as the complex mechanisms of drug resistance, it is necessary to carry out a variety of immunotherapy-combined regimens to improve the efficacy and reduce or overcome the drug resistance of advanced GC. Current combination strategies include different immunotherapy with chemotherapy, anti-HER-2-targeted therapy, anti-angiogenesis therapy, and immunotherapy.

### Immunotherapy in Combination With Chemotherapy

#### Cancer Vaccine Combined With Chemotherapy

DC vaccines have been used to stimulate immunity in the treatment of cancer patients. In a phase II study with metastatic or unresectable GC/GEJ adenocarcinoma, the treatment of gastrin-17 diphtheria toxoid (G17DT) vaccine combined with chemotherapy [cisplatin þ 5-fluorouracil (5-FU)] resulted in a long time to progression (TTP) and longer OS in 69% of patients (39). A study of vascular endothelial growth factor receptor (VEGFR) 1 and 2 vaccine combined with S-1/cisplatin in metastatic or recurrent gastric adenocarcinoma showed its usefulness with an ORR and disease control rate (DCR) of 55 and 100%, an OS of up to 14.2 months, and a 1- and 2-year survival of 68.2 and 25.9% (40). The lack of antigenicity and the failure to provide adequate co-stimulation, as well as the inactivation of T cells against tumors, are likely leading to the poor efficacy of cancer vaccines (41). A clinical trial evaluated the outcome of patients that received vaccine plus chemotherapy or chemotherapy alone. Disease free survival (DFS) was higher in the group that received vaccination (HSP gp96 vaccination) (*p* = 0.045), and 2-year OS was 81.9 vs. 67.9% (*p* = 0.123) in the vaccination plus chemotherapy and chemotherapy alone groups, respectively (42). Moreover, due to the characteristic of HLA being restricted, RNA vaccines become a novel option in cancer immunotherapy and are therefore safer and well-tolerated by cancer patients (43). As such, there are an increasing number of researchers giving attention to RNA vaccines.

#### Adoptive Cell Therapies Combined With Chemotherapy

A study evaluated ACT with TILs in stage IV GC patients divided into chemotherapy-only or ACT plus chemotherapy groups. The combination group showed a higher OS and 50% survival rates compared to the chemotherapy group (11.5 vs. 8.3 months). However, the survival benefit was not associated with OR in this trial (44). Another clinical trial evaluated the efficacy of ACT (cells cultured with cytokines and anti-CD3) plus chemotherapy in 151 stage III/IV GC patients in the adjuvant setting. Although 5-year OS was not significantly different, the 5-year DFS was significantly increased in the combination group (28.3% vs. 10.4%) (45). The investigators used autologous natural killer (NK) cells, γδ T cells, and CIK cells in combination with chemotherapy to treat patients with advanced GC and found that the combination group had better prognosis and tolerability, and lower disease recurrence rate than the group treated with chemotherapy alone (46). The results of a meta-analysis of chemotherapy combined with DC-CIK for advanced GC showed that the DCR, ORR, and quality of life were significantly higher in the combination group; in addition, the levels of CD3, CD4, CD3, CD56, IFN-γ, and IL-12 related to immune function detected in the blood were significantly higher than those in the chemotherapy-alone group (47). The existing clinical trial data suggest that the responses of GC to ACTs are encouraging, but there are an inadequate number of ongoing clinical trials.

#### ICIs Combined With Chemotherapy

Keynote-059 cohort 2 and cohort 3 (48) studied the first-line treatment of advanced GC with pembrolizumab alone or in combination with chemotherapy. Cohort 2 showed that the results of the combination group were significantly better than those for monotherapy, especially in the PD-L1^+^ group. Cohort 3 included only PD-L1^+^ patients, with an overall ORR of 26%, DCR of 36%, mPFS of 3.3 months, and mOS of 20.7 months. The interim data of the ATTRACTION-04 trial (49) showed that ORR of patients receiving nivolumab/SOX or nivolumab/CapeOX ware 57.1 and 76.5%, respectively. Furthermore, the mOS was not reached in both groups, and most of grade ≥ 3 TRAEs were common side effects of chemotherapy, as expected for follow-up results. Thus, the combined use of ICIs and chemotherapy in GC preliminarily showed better effect than that of monotherapy, and adverse events were mainly related to chemotherapy and were tolerable, which promote the development of multiple large, phase III clinical trials to assess its efficacy more effectively and accurately. The ongoing phase 3 trial evaluating combination chemotherapy with checkpoint inhibitors as a first-line treatment in PD-L1^+^/HER-2^−^ advanced GC is Keynote-062 **(NCT02494583)**, which is divided into three groups, pembrolizumab, pembrolizumab in combination with cisplatin/5-FU, and cisplatin/5-FU alone. The Phase III Checkmate-649 study with a larger sample size is exploring the efficacy and safety of nivolumab combined with XELOX or FOLFOX chemotherapy vs. first-line chemotherapy alone for advanced GC/GEJC **(NCT02872116)**. The phase II Keynote-659 trial is evaluating the safety and efficacy of pembrolizumab combined with chemotherapy as a first-line treatment for advanced GC **(NCT03382600)**. At present, the efficacy of immunotherapy combined with chemotherapy in the treatment of GC still needs to be evaluated continuously. In the future, we should fully consider the particularity of the immune microenvironment of GC and explore new combination therapy strategies.

### Immunotherapy in Combination With Antiangiogenic Agents

Preclinical studies suggest that VEGF inhibited by antiangiogenic agents has immunomodulatory activity, which provides a rationale for their use with ICIs (50). In a study of pembrolizumab combined with ramucirumab (anti-VEGFR-2) in gastroesophageal cancer, ORR and OS of PD-L1^+^ patients were 9% and 14.9 months, respectively, while the results of patients who were PD-L1– were only 6% and 5.2 months (51). A phase I trial in 69 patients with advanced GC/GEJC studied the efficacy and safety of pembrolizumab plus ramucirumab as first-line and second-line or later subgroups. The results showed that ORR was 14 and 7%, and grade ≥ 3 TRAEs were 39 and 27%, respectively (52), supporting the additive for ramucirumab to ICIs. Other ongoing trials of ICIs plus antiangiogenic agents include trials of atezolizumab plus bevacizumab with or without chemotherapy **(NCT01633970)**, nivolumab plus ramucirumab **(NCT02999295)**, pembrolizumab plus ramucirumab **(NCT02443324)**, and durvalumab plus ramucirumab **(NCT02572687)**.

### Immunotherapy in Combination With Anti-HER-2 Antibody and Chemotherapy

Currently, the first-line standard treatment for advanced HER-2^+^ advanced GC/GEJC adenocarcinoma is trastuzumab combined with chemotherapy. HER-2 overexpression has been shown to suppress the immune response within the tumor microenvironment. Inhibition of HER-2 can promote T cell activation and transport, enhance NK cells to produce IFN-γ, and enhance the ADCC effect. Thus, combination therapy of an anti-HER-2 monoclonal antibody and a PD-1/PD-L1 inhibitor may have synergistic effects (53). In patients with HER-2^+^ metastatic EG cancer, first-line treatment with the combination of pembrolizumab and trastuzumab plus chemotherapy showed encouraging clinical activity (54). A phase II clinical trial is ongoing to evaluate the effectiveness and tolerability of pembrolizumab in combination with HER-2 antibody margetuximab **(NCT02689284)** and trastuzumab **(NCT02901301)** (55). The phase III Keynote-811 study exploring the effect of adding pembrolizumab to chemotherapy and trastuzumab is still in its enrollment phase **(NCT036153260)**. A phase I/II trial involving various cancers including GC with the treatment of NK cells plus trastuzumab is in its recruitment phase **(NCT02030561)**.

### Dual Immunotherapy Combined Strategies

Preclinical data showed that blocking both PD-1 and CTLA-4 signal transduction can increase IFN-γ production by lymphocytes, increase the expression of CD4/CD8 on TILs, and reduce Tregs in tumors to increase antitumor activity. The Checkmate-032 study (56) explored the efficacy of nivolumab alone or in combination with ipilimumab (different dosage) in second- and third-line treatments of advanced GC/GEJC in the Western population. Although both ORR and mOS were the best in the N1 + I3 (nivolumab 1 mg/kg + ipilimumab 3 mg/kg Q3W) group, its side effects cannot be ignored. 47% grade 3/4 irAEs were observed in the nivolumab/ipilimumab group of the phase III CheckMate 649 study **(NCT03215706)**, making it difficult to combine this regimen with chemotherapy. Thus, the main obstacle and limitation of the immunotherapy-combined treatment of GC is the increased high frequency and severity of irAEs (57). Almost all patients (93%) had irAEs after concurrent combination therapy with anti-PD-1 and anti-CTLA-4, with grade 3 or 4 irAEs increasing (50%). In melanoma trials, high-grade irAEs were 21% with anti-PD-1 monotherapy (nivolumab), 28% with anti-CTLA-4 monotherapy, and 59% with the combination of anti-CTLA-4 and anti-PD-1 (58). IrAEs usually involve the gastrointestinal tract, lungs, skin, endocrine glands, and liver and less frequently involved central nervous system and cardiovascular, musculoskeletal, and hematological systems. Still, a phase I/IIb study of durvalumab in combination with tremelimumab for gastric adenocarcinoma is ongoing to explore in depth **(NCT02340975)**.

### Immunotherapy in Combination With Other Therapeutic Strategies

In addition to CTLA-4 and PD-1/PD-L1, inhibitors of other immune checkpoint proteins [T cell immunolobulin and mucin-con-taining protein-3 (TIM3), lymphocyte activation gene 3 (LAG3)], co-stimulatory receptors expressed on T cells [glucocorticoid-induced tumor necrosis factor receptor–related protein (GITR), OX40, 4-1BB], enzymes indoleamine 2,3-dioxygenase (IDO-1), etc. (59) may synergize with anti–PD-1/PD-L1 inhibitors to generate a more robust antitumor immune response. Trials examining these strategies in EG cancer and various other cancers include nivolumab plus BMS-986016 (anti–LAG-3; **NCT01968109**) and pembrolizumab plus epacadostat (IDO-1 inhibitor; **NCT02178722 and NCT03196232)**. In addition, the FRACTION-GC study is assessing nivolumab plus LAG-3 inhibitor (BMS-986016) or ipilimumab specifically in patients with advanced GC **(NCT02935634)**. The therapeutic regimen of anti–GITR agent (INCAGN01876) and nivolumab combined with or without ipilimumab is being investigated in advanced tumors with a cohort of patients with advanced GC/GEJC **(NCT03126110)**. In addition, matrix metalloproteinase 9 (MMP9) is a protein that is overexpressed in many solid tumors. It could remodel the extracellular matrix and is related to the recruitment of angiogenesis and myeloid suppressor cells and regulatory T cells. A trial is investigating a combination of nivolumab and MMP9 inhibitor GS-5745 in patients with unresected or relapsed GC/GEJC adenocarcinoma **(NCT02864381)**. Furthermore, phase I/II trials of ICIs plus other molecules like INCB054828, a pan-inhibitor of Fibroblast growth factor receptor (FGFR) types 1, 2, and 3, are ongoing **(NCT02393248)**. Another trial is studying a combination of pembrolizumab and CRS-207, a live attenuated *Listeria monocytogenes* vaccine genetically engineered to overexpress mesothelin for patients with advanced GC/GEJC **(NCT03122548)**.

Ongoing trials of novel combination therapies not mentioned above are listed in Table 2.

**Table 2:** Ongoing trials of novel combination therapies.

**Clinical Trials.gov identifier**	**Intervention used**	**Phase**	**Estimated sample size**	**Population**	**Primary endpoints**
NCT02335411	Pembrolizumab (treatment naïve) OR pembrolizumab (previously treated) OR P+ cisplatin+ 5-FU+ capecitabine (treatment naïve); 1 line or more	Phase II	316	Advanced gastric and GEJ cancer	Adverse events; discontinuing study due to AE;ORR
NCT02318901	Pembrolizumab OR P+ ado-trastuzumab etamine OR P+ cetuximab	Phase Ib/II	90	Patients with advanced cancer (one cohort for patients with unresectable HER-2+ gastric or GEJ cancers)	Recommended phase 2 dose of trastuzumab with pembrolizumab
NCT02658214	Durvalumab+ 5-FU+ oxaliplatin + leucovorin; 1 line	Phase I	60	Cohort 5 for advanced GC/GEC	Safety/tolerability of first line therapy; Incidence of adverse events
NCT02746796	ONO-4538+ SOX (Part 1) ONO-4538+ Cape OX (Part 1) ONO-4538+ Chemo group (Part 2) → either SOX or Cape OX Placebo+ Chemo group (Part 2); 1 line	Phase II	680	Unresectable advanced or recurrent gastric and GEJ cancer	PFS;OS
NCT02572687	MEDI4736 in combination with ramucirumab	Phase I	114	Locally advanced and unresectable or metastatic gastrointestinal or thoracic malignancies including gastric or GEJ adenocarcinoma	DLTs
NCT02268825	MK-3475 (pembrolizumab) in combination with mFOLFOX6	Phase I/IIa	128	Various advanced gastrointestinal Cancers	Safety of combination of FOLFOX and MK-3475
NCT02903914	INCB001158 (CB-1158) alone or in combination with Pembrolizumab (advanced/metastatic gastric and GEJ cancer that have never received prior checkpoint inhibitor therapy)	Phase I/II	424	Various advanced/metastatic solid tumors including GC	Safety, pharmacokinetics; biomarkers and tumor response.
AIO-STO-0217 (NCT03409848)	(nivolumab + trastuzumab) in combination with FOLFOX vs. ipilimumab; 1 line	Phase II	Recruiting	Previously untreated HER-2+ locally advanced or metastatic esophagogastric adenocarcinoma.	OS

*DLTs, dose-limiting toxicity; 5-FU, 5-fluorouracil; GC, gastric cancer; GEJ, gastric or gastroesophageal junction; GC/GEJC, gastric or gastroesophageal junction cancer; ORR, objective response rate; AE, adverse event; OS, overall survival; PFS, progression free survival; HER-2, Human epidermal growth factor receptor-2*.

## Identifying Prognostic and Predictive Biomarkers for Immunotherapy in GC/GEJC

Currently, PD-1/PD-L1 inhibitors are approved as a third-line treatment for PD-L1^+^ and MSI-H refractory metastatic gastroesophageal cancer (25). However, from the research data, regardless of PD-L1 expression levels, the ORR of immunotherapy applied to end-line treatment for GC is less than 20%. With such low ORR, it is necessary to explore predictive biomarkers in the future to identify patients who would benefit from immunotherapy for gastroesophageal cancer. At present, PD-L1 expression and MSI-H/mismatch repair deficiency (dMMR) have been recognized and have become common markers for predicting efficacy in the clinical setting (25), but there still exist many limitations in the effective and accurate evaluation of patient efficacy and prognosis. EBV infection, tumor mutation burden (TMB), and the search for new biomarkers are currently potential research directions. There has been a greater understanding of the complex dynamics of the immune signaling necessary for antitumor responses. As such, the application of multiple immunomarkers to evaluate immune gene expression profiles, comprehensive immune scores, and tumor microenvironment phenotypes have entered into the forefront of biomarker analyses, providing insights into the molecular characteristics of response to immunotherapy and greater specificity in predicting efficacy. The two important biomarkers are detailed below.

### PD-L1 Expression

Studies have shown that PD-L1 is expressed in 30–65% invasive GCs and is related to the depth of tumor invasion, lymph node metastasis, distant metastasis, tumor size, EBV infection, etc., which is a negative marker of prognosis (60–62). Currently, FDA has an approved PD-L1-positive expression as a biomarker for third-line treatment of pembrolizumab in gastric cancer (24), and many regions had approved nivolumab for the treatment of unresectable advanced or recurrent GC regardless of PD-L1 expression. In addition, the correlation between PD-L1 expression and efficacy of nivolumab appears to be related to race. In the ATTRACTION-2 phase III study (28) in the Asian population, ORR of nivolumab monotherapy was 11% and 12-month OS rate increased to 27%, and this survival benefit was not related to PD-L1 expression, while in the CheckMate-032 study (56) in Western patients, the ORR rate in PD-L1^+^ tumors was significantly higher than in negative tumors (27 vs. 12%). At present, the PD-L1 level as a predictive biomarker for anti-PD-1/PD-L1 therapy in clinical trials still has many problems. For example, the definition of PD-L1^+^GC/GEJC is based on a comprehensive positive score, including the expression on tumor cells, lymphocytes, and macrophages, which is different from the definition in lung cancer (25); there is still no consensus on the cutoff value of PD-L1-positive expression, and the expression of PD-L1 was affected by many factors such as standardization of measurement methods, antitumor therapy, and immune response of the host.

### Tumor Mutation Load

TMB is a powerful predictor of response to ICIs in multiple tumor types. Clinically, next-generation sequencing can be used to capture the TMB of malignant tumors. Li et al. (63) used the Foundation One platform for sequencing and defined high TMB as >20 mut/Mb, which was found only accounting for 5% of 1,485 cases of GC. An earlier report by Licitra et al. (64) suggested that TMB ≥ 14 mut/Mb would benefit more from immunotherapy (2-year OS rate was 15 vs. 60%, *p* = 0.094). However, the proportion of patients with this high TMB subset was small (6/55), 4 of which were dMMR tumors. The follow-up report of the IMPACT team on gastroesophageal cancer seems to indicate that a cutoff value of >9.7 mut/Mb of TMB represents the top quartile of 40 patients treated with ICIs, which is more relevant to clinical benefit (mOS is 16.8 vs. 6.62 months, *p* = 0.058) (65). Therefore, further research is needed to determine if there is an ideal cutoff value of TMB and evaluate the predictive efficacy of TMB in GC.

## Safety of Immunotherapy in GC/GEJC

Because of their immunological mechanism of action, adverse effects of immunotherapies are distinctive from those of conventional chemotherapies. Overall, the safety of immunotherapy in GC/GEJC was better than that of chemotherapy (grade 3–5 TRAE was 35 vs. 14%) (26). Cancer vaccines are associated with minimal toxicities. Common adverse effects are similar to those associated with vaccination against pathogens such as induration, fatigue, fever, and chills (15). For ACTs, the adverse effect profiles are less well-defined with major AEs including on-target off-tumor toxicities similar to those observed in autoimmune diseases, which result from the sharing of antigens between tumor and healthy cells. In general, ACTs are associated with a benign AE profile that ranges from mild to moderate constitutional symptoms in GC. As for checkpoint inhibitor therapies, the side effects are roughly similar with about 10–20% of grade 3 or higher, involving fatigue, pruritis, arthralgias, diarrhea, and elevated aminotransferases (66). Also due to the activated effects of preexisting autoreactive T cells and B cells, these therapies can lead to dermatitis, pneumonitis, colitis, and hepatitis as well as endocrinopathies (67), with pneumonia and colitis being the most common grade 3 irAEs in GC patients. Immunotherapy can also lead to more severe complications as a result of their immune-related effects. For example, neurotoxicity (linked to the release of IL-2) and cytokine release syndrome (linked to the release of IL-6, IFN-γ, and TNF-α) induced by ACTs are potentially fatal if not diagnosed in a timely manner. Compared to PD-1/PD-L1 monotherapy, anti-CTLA-4 antibodies, and combined regimens have a higher incidence of TRAEs (68). Further research and better characterization are needed as serious and fatal toxicities have been reported with the use of immunotherapy in other cancers.

## Summary and Outlook

In recent years, immunotherapies involving cancer vaccines, adoptive cell therapies, and ICI therapies have gradually been developed and advanced into novel frontiers of treatment for advanced GC/GEJC, revolutionizing the therapeutic landscape. The development of immune combination therapies, identification of irAEs, and search for more robust predictive biomarkers are essential for improving the treatment efficacy of patients with advanced GC/GEJC and overcoming the drug resistance problem.

There are still many challenges in immunotherapy of advanced GC/GEJC, which are also future directions that need in-depth study. Firstly, in which stage of advanced tumors should we use immunotherapy in earlier lines or after disease progression with more than two lines of therapy? We look forward to the ongoing phase III trials and wait with hope for their results. Two studies carried out in our study center have confirmed the efficacy of immunotherapy combined with chemotherapy in the treatment of stage III GC (69, 70), suggesting that the clinical application of immunotherapy may be expanded to early-stage GC. Moreover, considering that only a minority of patients with ICIs can achieve a durable response, multimodal treatment strategies in addition to combination therapy should be developed to improve patient clinical outcomes and overcome the development of resistance. Insights into specific molecular subtypes and genomic alterations could prompt the development of more precise novel therapies in the future. Secondly, the complex resistance mechanisms to immunotherapy are still not well-understood. The gradual elucidation and in-depth exploration of new immune resistance mechanisms contribute to the discovery of new therapeutic targets and continue to expand the scope of clinical applications of cancer immunotherapy. Additionally, more studies are needed to confirm predictive and prognostic biomarkers to immunotherapy agents in GC. However, due to the complexity of the antitumor immune response and tumor heterogeneity among different patients, there are currently no suitable wide and uniform biomarkers to predict clinical benefits. Nevertheless, this exploration can help screen immunotherapy-dominant populations, develop personalized precise diagnosis and treatment programs, predict the efficacy of treatment, and adjust the treatment regimen in a timely manner. Finally, the toxicities and tolerability of these new combinations, especially dual immunotherapy-combined strategy, are important issues to be managed in these trials. In future studies, exploring biomarkers of irAEs is an area that should be focused, which relies on the constant revelation of their mechanisms. Predictors associated with irAEs should be comprehensively analyzed and identified and reduce the incidence and severity of irAEs through early intervention, or timely detection and treatment, which facilitates the continuous optimization of clinical decision-making and patient care and the achievement of maximum clinical benefit.

In conclusion, much progress has been achieved in the treatment of advanced GC/GEJC over the past decade. With the recent molecular and biologic exploration, we have recognized that GC is a group of distinct molecular entities rather than a single disease. It is unquestionable that this field is moving to more precise medicine, and constant accomplishments will transform the management of advanced GC/GEJC in the clinical setting in the near future.

## Author Contributions

RB reviewed the literature, analyzed, and wrote the paper. NC, TL, LL, and ZL consulted the literature, reviewed, and modified the article. XL and JC put forward valuable comments on the article, reviewed, and edited it. All authors read and approved the final manuscript.

## Conflict of Interest

The authors declare that the research was conducted in the absence of any commercial or financial relationships that could be construed as a potential conflict of interest.

## Abbreviations

ACT: adoptive cell therapy
CAR: chimeric antigen receptor
CEA: carcinoembryonic antigen
CIK: cytokine-induced killer
CIN: chromosomal instability
CTA: cancer-testis antigen
CTLA-4: cytotoxic T-lymphocyte-associated protein 4
CPS: combined positive score
DC: dendritic cell
DCR: disease control rate
DFS: disease free survival
dMMR: mismatch repair deficiency
EBV: Epstein-Barr virus
EAAL: expanded activated autologous lymphocyte
EpCAM: epithelial cell adhesion molecule
FDA: Food and Drug Administration
FGFR: Fibroblast growth factor receptor
5-FU: 5-fluorouracil
GC: gastric cancer
GC/GEJC: gastric or gastroesophageal junction cancer
GITR: glucocorticoid-induced tumor necrosis factor receptor–related protein
G17DT: gastrin-17 diphtheria toxoid
HSP: heat shock proteins
HER-2: Human epidermal growth factor receptor-2
HLA: human leukocyte antigen
ICI: immune checkpoint inhibitor
IDO-1: indoleamine 2,3-dioxygenase
irAEs: immune-related adverse events
LAG3: lymphocyte activation gene 3
MSI: microsatellite instability
MAGE-3: melanoma-associated antigen 3
MMP9: matrix metalloproteinase 9
NK: natural killer
OS: overall survival
ORR: objective response rate
PFS: progression free survival
PD-1: programmed death-1
PD-L1: programmed death-ligand 1
SD: stable disease
TTP: time to progression
TRAE: treatment related adverse event
TCGA: the Cancer Genome Atlas
TMB: tumor mutation burden
TIL: tumor infiltrating lymphocyte
TIM3: T cell immunolobulin and mucin-con-taining protein-3
VEGFR: vascular endothelial growth factor receptor.
